# Erbium-doped fiber laser passively mode locked with few-layer WSe_2_/MoSe_2_ nanosheets

**DOI:** 10.1038/srep23583

**Published:** 2016-03-24

**Authors:** Dong Mao, Xiaoyang She, Bobo Du, Dexing Yang, Wending Zhang, Kun Song, Xiaoqi Cui, Biqiang Jiang, Tao Peng, Jianlin Zhao

**Affiliations:** 1 Shaanxi Key Laboratory of Optical Information Technology, School of Science, Northwestern Polytechnical University, Xi’an 710072, China; 2 Key Laboratory of Space Applied Physics and Chemistry, Ministry of Education, School of Science, Northwestern Polytechnical University, Xi’an 710072, China

## Abstract

Few-layer transition-metal dichalcogenide WSe_2_/MoSe_2_ nanosheets are fabricated by a liquid exfoliation technique using sodium deoxycholate bile salt as surfactant, and their nonlinear optical properties are investigated based on a balanced twin-detector measurement scheme. It is demonstrated that both types of nanosheets exhibit nonlinear saturable absorption properties at the wavelength of 1.55 μm. By depositing the nanosheets on side polished fiber (SPF) or mixing the nanosheets with polyvinyl alcohol (PVA) solution, SPF-WSe_2_ saturable absorber (SA), SPF-MoSe_2_ SA, PVA-WSe_2_ SA, and PVA-MoSe_2_ SA are successfully fabricated and further tested in erbium-doped fiber lasers. The SPF-based SA is capable of operating at the high pump regime without damage, and a train of 3252.65 MHz harmonically mode-locked pulses are obtained based on the SPF-WSe_2_ SA. Soliton mode locking operations are also achieved in the fiber laser separately with other three types of SAs, confirming that the WSe_2_ and MoSe_2_ nanosheets could act as cost-effective high-power SAs for ultrafast optics.

Two-dimensional layered materials have attracted increasing attention from fundamental researches to industrial applications attributing to their unique dimensionality effect as well as outstanding physical/chemical property^1^,^2^,^3^,^4^. Graphene, the most famous two-dimensional material, possesses a Dirac-like electronic band structure, enabling ultra-broadband response ranging from ultraviolet to terahertz^5^. Since the few- and single-layer graphene was fabricated by the micromechanical cleavage method, it has been in-depth studied and applied in fields of photon detection, graphene plasmonics, ultrafast optics, quantum electrodynamics, *et al.*^6^,^7^,^8^,^9^. However graphene also suffers the deficiency of the weak absorption coefficiency (2.3% of incident light per layer), which limits its modulation ability and potential applications. In addition to graphene, transition-metal dichalcogenides (TMDs), a family of inorganic two-dimensional layered materials with the chemical formula of MX_2_, where M is a transition metal (usually Mo, W) and X is a group VI element (S, Se, Te), are attracting continuously rising interest due to its exotic physical properties such as non-zero bandgap and layer-dependent second-order optical nonlinearity^10^,^11^,^12^,^13^. The in-plane atoms of TMD materials are held together by strong chemical bonds while weak Van der Waals interaction enables stacking between layers, which enables them to be exfoliated into thin nanosheets for developing high-performance optoelectronic devices^14^,^15^.

Molybdenum disulfide (MoS_2_) and tungsten disulfide (WS_2_), two representative TMDs, received the most research attention during the past several years^16^,^17^,^18^. In the branch of optics, a great deal of research interest is focused on the nonlinear saturable absorption property of MoS_2_ and WS_2_ nanosheets^19^,^20^,^21^,^22^,^23^. Wang *et al.* reported that few-layer MoS_2_ display ultrafast saturable absorption property at 515 nm and 800 nm^20^, and observed the strong two-photon absorption and saturable absorption respectively in mono- as well as multi-layer MoS_2_ at 1030 nm^24^. By the introduction of sulfur defects in MoS_2_, Yu *et al.* found that the bandgap of MoS_2_ nanosheets can be significantly reduced, and realized a broadband saturable absorber (SA) achieving Q-switched lasers at 1.06, 1.42, and 2.1 μm^21^. Recently, several groups including us demonstrated that WS_2_ nanosheets exhibit broadband saturable absorption property even at sub-bandgap photon energies^25^,^26^. Hereafter, various types of MoS_2_ or WS_2_ SAs were proposed to realize passively mode-locked or Q-switched lasers^26^,^27^,^28^,^29^.

In comparison with MoS_2_ and WS_2_, molybdenum diselenide (MoSe_2_) and tungsten diselenide (WSe_2_) exhibit the similar molecular structure while have smaller optical bandgaps. For instance, the bandgaps of bulk MoS_2_ and WS_2_ are 1.29 and 1.35 eV while that of MoSe_2_ and WSe_2_ are 1.09 and 1.2 eV respectively^30^. This special bandgap is particularly interesting for laser applications from the visible to near-infrared wavelength regime^31^,^32^,^33^. Luo *et al.* demonstrated a compact red-light Q-switched praseodymium-doped all-fiber laser using two-dimensional TMDs as SAs^34^. However, the study of WSe_2_ and MoSe_2_ is mainly concentrated on fabrication method and electronic property^32^,^35^ and their nonlinear optical behavior still remains less addressed up till now. We think that the investigation of emerging saturable absorption material is a key step for developing its potentials. In this contribution, we prepare WSe_2_ and MoSe_2_ nanosheets with a liquid exfoliation method using sodium deoxycholate bile salt as surfactant. Experimental results demonstrate that both WSe_2_ and MoSe_2_ nanosheets display saturable absorption property at the wavelength of 1.55 μm. By utilizing SPF- and PVA-based WSe_2_/MoSe_2_-SAs, stable picoseconds soliton pulses are delivered from the erbium-doped fiber (EDF) laser separately, including a train of 3252.65 MHz harmonically mode-locked ultrafast pulses.

## Results

### Preparation and characterization of WSe_2_/MoSe_2_ SAs

Various techniques have been explored to prepare mono- or few-layer TMD nanosheets, such as chemical vapor deposition, micromechanical exfoliation, liquid exfoliation, *et al.*^11^. Each method has its advantages and ranges of applications. For example, micromechanical exfoliation is capable of producing high-quality nanosheets, while the production efficiency and size are limited. Chemical vapor deposition could synthesize large-scale mono- or few-layer TMD films. However, the synthetic process is quite complicated and requires high temperature. Liquid exfoliation is a simple and cost-effective method to produce mass dispersions of mono- and few-layer TMD nanosheets or other two-dimensional materials at ambient conditions^36^,^37^. Here, the WSe_2_/MoSe_2_ nanosheets are prepared by the liquid exfoliation method, which is similar to that of previous reports^36^.

**Figure 1:**
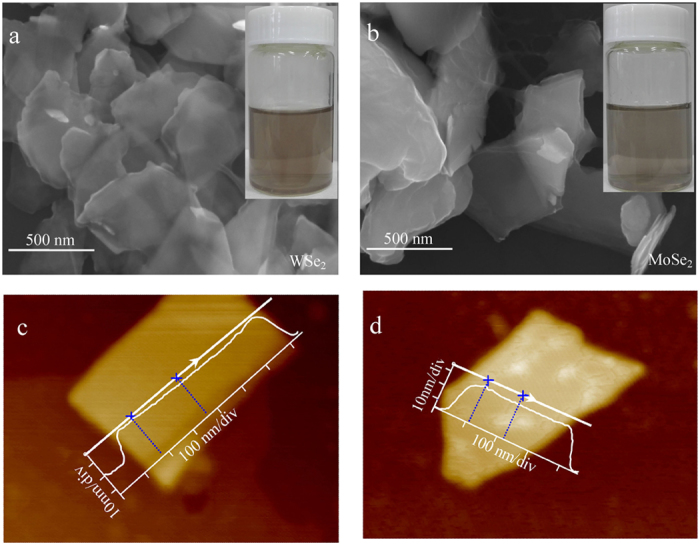
Characterization of WSe_2_/MoSe_2_ nanosheets. SEM images (**a,b**) and AFM images (**c,d**). The inset of Fig. 1(a,b) shows the corresponding suspensions of the nanosheets.

The insets of Fig. 1(a,b) show the suspensions of WSe_2_/MoSe_2_ nanosheets, which display faint red color for WSe_2_ and slight grey for MoSe_2_ respectively. These suspensions are quite stable, and display no aggregation after being stored for several weeks under ambient conditions. The nanosheets are characterized by scanning electron microscope (SEM), as illustrated in Fig. 1(a,b). Both WSe_2_ and MoSe_2_ are present as two-dimensional nanosheets with intact surface texture, confirming successful exfoliation of the bulk-state crystal. The length and width of most nanosheets are below 1 μm, which depends on the centrifugation rate during the fabrication. The atomic force microscope (AFM) image illustrate that the thickness of nanosheets is ~20 nm for WSe_2_ and ~15 nm for MoSe_2_, as illustrated in Fig. 1(c,d). Based on above results, one can convince that the WSe_2_/MoSe_2_ nanosheets are successfully fabricated using the proposed method.

**Figure 2:**
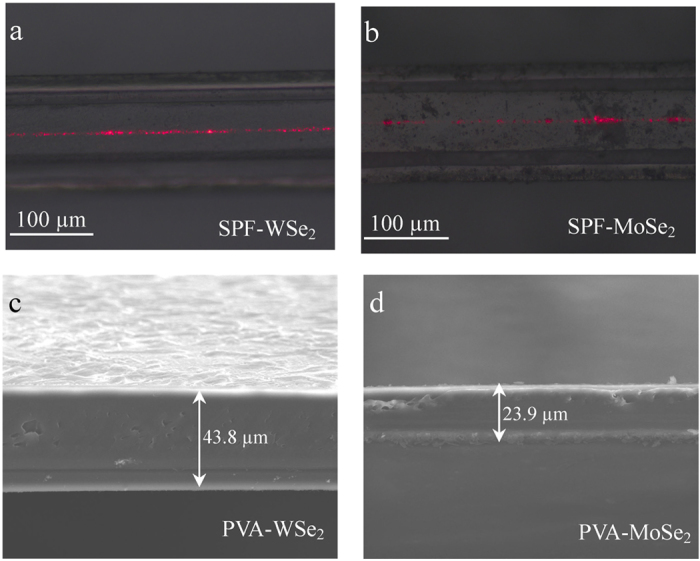
Optical micrograph of SPF deposited with WSe_2_ nanosheets (**a**) and MoSe_2_ nanosheets (**b**). Side profile of WSe_2_-PVA film (**c**) and MoSe_2_-PVA film (**d**).

After the successful exfoliation of the bulk crystal, we then fabricate two types of WSe_2_/MoSe_2_-based SAs and investigate their saturable absorption properties. The first type of SA is obtained by depositing the WSe_2_/MoSe_2_ nanosheets onto a side polished fiber (SPF) to realize mutual interaction of nanosheets with the evanescent field of light within the SPF. The SPF is fabricated by polishing a bent single-mode fiber (SMF) on one side. The depth between the polished surface and fiber core of SPF is about 2 μm, and the polished part of the fiber has a length of 1.2~1.5 mm. Then the WSe_2_/MoSe_2_ nanosheets dispersion is dropped on the surface of SPF that is fixed on a quartz plate. At last, a 10 mW 1.55 μm continuous wave is coupled into the SPF and the optical deposition process starts. The deposited length and depth depend on the deposition time and input power, and the insertion loss is monitored in real time with an optical power meter. Actually, the insertion loss of the SA should be moderate, for example, mode locking operation can be achieved when the insertion loss ranges from 20% to 80%. By virtue of lateral interaction scheme, the TMD nanosheets interact with light over long distance while experience low intensity, which allows the SA to work at the high-power regime. Figure 2(a,b) shows the SPF deposited with WSe_2_/MoSe_2_ nanosheets after injecting a 632.8 nm He-Ne laser. The red laser is scattered from the surface of the SPF, indicating the existence of evanescent light field.

The other type of SA is fabricated by mixing the WSe_2_/MoSe_2_ nanosheets with aqueous PVA solution and evaporating the mixture on a substrate. This process could be described as follows. First, 5 Wt% PVA solution and the WSe_2_/MoSe_2_ dispersions are mixed at a volume ratio of 1:3 by a magnetic stirrer for 5 hours. Then, a thin WSe_2_/MoSe_2_-PVA film is formed by evaporating the mixture on a quartz plate under ambient temperature and pressure for 48 hours. At last, the film is cut into small pieces (~1 × 1 mm) and sandwiched between two facets of a fiber connector to realize fiber-based SA. The thicknesses of WSe_2_/MoSe_2_-PVA film are measured as 43.8 and 23.9 μm, as shown in Fig. 2(c,d), respectively. Compared with optical deposition^38^ or dropping nanosheets on quartz plate^39^, the PVA-film method would be more attractive due to its advantages of controllability, flexibility, and cost-effectiveness.

**Figure 3:**
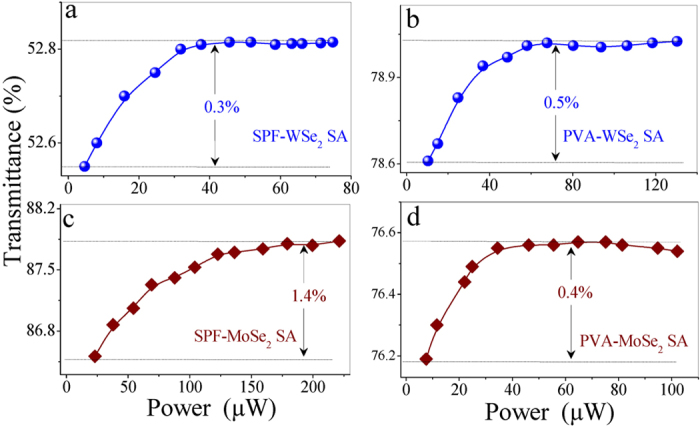
Nonlinear transmissions of SPF-WSe_2_ SA (**a**), PVA-WSe_2_ SA (**b**), SPF-MoSe_2_ SA (**c**), and PVA-MoSe_2_ SA (**d**).

Based on a balanced twin-detector measurement method^40^, we investigate the nonlinear saturable absorption property of four SAs (SPF-WSe_2_, PVA-WSe_2_, SPF-MoSe_2_, and PVA-MoSe_2_). Figure 3(a,d) shows the saturable absorption data of the WSe_2_/MoSe_2_ SA as a function of pulse power. One can observe that the transmission efficiencies of four SAs increase with pulse intensity, which is the typical characteristic of nonlinear saturable absorption. The modulation depths of SPF-WSe_2_ and PVA-WSe_2_ SAs are given as 0.3% and 0.5%, and that of the MoSe_2_ are given as 1.4% and 0.4%. During the experiment, we did not observed nonlinear response from other devices, which suggest that the saturable absorption property is purely caused by TMD nanosheets. The proposed SAs may find wide applications in ultrafast photonics, including high-speed light modulation, optical switching, and ultrashort pulse generation.

### Setup of WSe_2_/MoSe_2_ mode locked fiber laser

**Figure 4:**
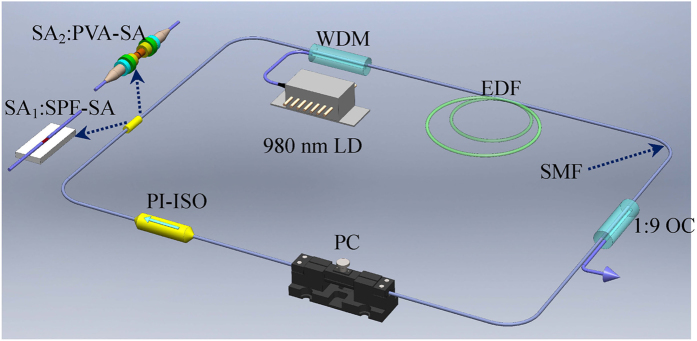
Schematic of the passively mode-locked fiber laser based on WSe_2_/MoSe_2_ SAs. SA_1_ and SA_2_ are SPF- and PVA-based SAs, respectively. Wavelength division multiplexer (WDM); Erbium-doped fiber (EDF); Output coupler (OC); Single-mode fiber (SMF); Polarization controller (PC); Polarization insensitive isolator (PI-ISO).

Fiber lasers possess inherent advantages of alignment-free operation, excellent beam quality, high efficiency^41^,^42^, which provides an ideal platform to study the nonlinear optical property of new emerging materials such as carbon nanotube^40^,^43^, graphene^6^,^43^,^44^, topological insulator^39^, black phosphorus^45^,^46^, and TMDs^28^. Here, we construct an EDF laser to further test the saturable absorption property of four different SAs. The fiber resonator is composed of a 6 m EDF with 3 dB/m absorption at 980 nm, a fiber fused coupler with the output ratio of 10%, a polarization insensitive isolator, a SA, and a polarization controller, as shown in Fig. 4. The EDF used in the fiber laser is EDFC-980-HP, which has a mode field diameter of 5.8 ± 0.5 μm and doping concentration of 1500 ppm. A 980 nm laser diode with the maximum power of 610 mW is used to pump the EDF via a wavelength-division multiplexer. The polarization controller can adjust the polarization state of the laser, but it is not fundamental to realize the mode locking operation. The pigtails of WSe_2_ SA and MoSe_2_ SA are 0.7 and 2.8 m, respectively. The other fiber is SMF with a total length of 32 m. Considering the dispersion parameters *D* of −16 ps/(nm.km) and 17 ps/(nm.km) for EDF and SMF, the net dispersion *β*_2_ of the fiber laser is calculated as −0.59 ps^2^ for WSe_2_ SA and −0.63 ps^2^ for MoSe_2_ SA, which facilitates soliton pulse shaping through the interaction of self-phase modulation and anomalous group velocity dispersion.

### Experimental observations

**Figure 5:**
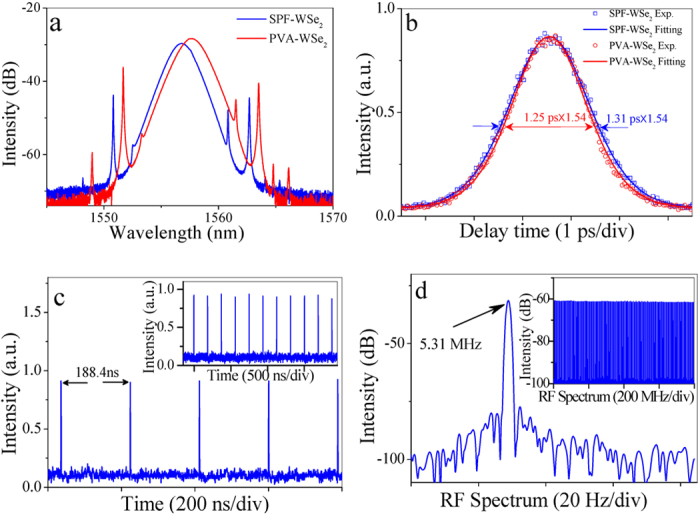
Output characteristics of WSe_2_ mode-locked fiber laser. (**a**) optical spectrum, (**b**) autocorrelation trace, (**c**) pulse train, and (**d**) RF spectrum. The inset shows the broadband RF spectrum at a span of 800 MHz.

We first investigate the mode-locking performance of the fiber laser by inserting the SPF-WSe_2_ SA into the cavity. At the pump power of 21 mW, self-starting soliton mode locking operation is achieved with the output power of 0.45 mW. As illustrated by the blue curve in Fig. 5(a), the output spectrum is centered at 1556.7 nm with a 3-dB bandwidth of 2 nm. Several pairs of sidebands is symmetrically distributed the optical spectrum, which is the typical characteristic of standard soliton. The pulse duration is measured by a high-sensitivity commercial autocorrelator. The autocorrelation trace has a full width at half maximum of 2 ps, as shown by the blue curve in Fig. 5(b). By using a Sech^2^ fitting, the pulse duration is estimated to be 1.31 ps. During the experiment, the fiber laser tended to operate at multiple-pulses state due to the combined effects of soliton energy quantization and long cavity length^47^. However, taking hysteresis phenomena of the soliton fiber laser, single-pulse mode locking can be obtained by slowly decreasing the pump power to 13 mW. The oscilloscope trace of the output pulse train is demonstrated in Fig. 5(c), which gives the pulse interval of 188.4 ns, well consistent with the cavity length of 38.7 m. The offset from zero position of y-axis on the oscilloscope trace may be attributed to the combined effects of weak input power and continuous wave of the sidebands. The fundamental repetition rate of the pulse is given as 5.31 MHz, as shown in Fig. 5(d). The inset shows the broadband RF spectrum at a span of 800 MHz. To verify whether the mode locking is induced by WSe_2_ nanosheets, we have replaced the SPF-WSe_2_ SA by the PVA-WSe_2_ SA in the cavity. In this case, soliton mode locking operation is also observed in the fiber laser at the pump of 35 mW. The optical spectrum and autocorrelation trace of the pulses are demonstrated by red curves in Fig. 5(a,b), respectively. Here, the output power of the fiber laser is measured as 0.84 mW, and the pulse evolution and radio frequency (RF) spectrum are quite similar with that of SPF-WSe_2_ mode-locked fiber laser, which are not present in the text for conciseness.

**Figure 6:**
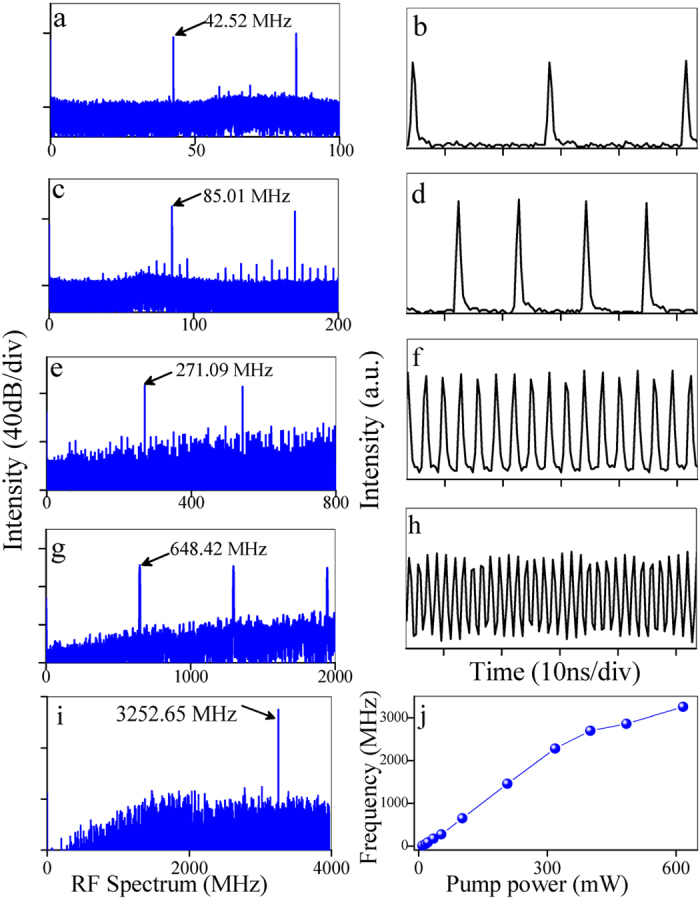
Harmonically mode-locked fiber laser based on the SPF-WSe_2_ SA. RF spectrum (**a,c,e,g,i**) and pulse train (**b,d,f,h**); (**j**) Frequency of output pulses as a function of the pump power.

Apart from the single- or multi-pulses operation reported previously, harmonic mode locking is also observed in the SPF-WSe_2_ mode locked fiber laser. We use a 27.5 GHz RF analyzer with a 45 GHz photodector to directly monitor the repetition rate of the output pulses. As shown in Fig. 6(a–i), by increasing the pump power from 25 to 610 mW, the repetition rate of the pulses gradually increases from 42.52 to 3252.65 MHz (612^th^ harmonic of fundamental repetition frequency), and the output power increases from 0.6 to 19 mW. Due to the bandwidth limitation (500 MHz), the 3252.65 MHz pulses cannot be displayed correctly on the used oscilloscope. Figure 6(j) summarizes the evolution of the pulse repetition rate as a function of the pump power. The repetition rate almost changes linearly at low pump power. Actually, the maximum pulse repetition rate is limited by the available pump power and can be further enhanced by optimizing the SA, such as using fiber-taper based SAs. The formation mechanism of harmonic mode locking can be attributed to long-period interaction between solitons^48^. When the solitons and non-soliton components exhibit certain phase difference, the interaction force becomes repulsive for all solitons within a pulse bunch and results in uniform distributed pulses inside the laser cavity. Such high-repetition-rate pulsed fiber laser can find important applications such as frequency combs and soliton communications.

As the extra heating can be rapidly dissipated from the SPF, the SPF-WSe_2_ SA can work at mode locking state at the pump power of 600 mW during the whole experiment (about four hours). Using bidirectional pump scheme with the total pump power of 1.2 W, the fiber laser can work at mode locking state for several minutes and then evolves into continuous wave state. In this case, the intracavity power is calculated as 400 mW from the output power. However, by decreasing the pump power to zero and then increasing pump power, mode-locking operation can be obtained again. Based on these results, we infer that the damage threshold of the SA is about 400 mW.

**Figure 7:**
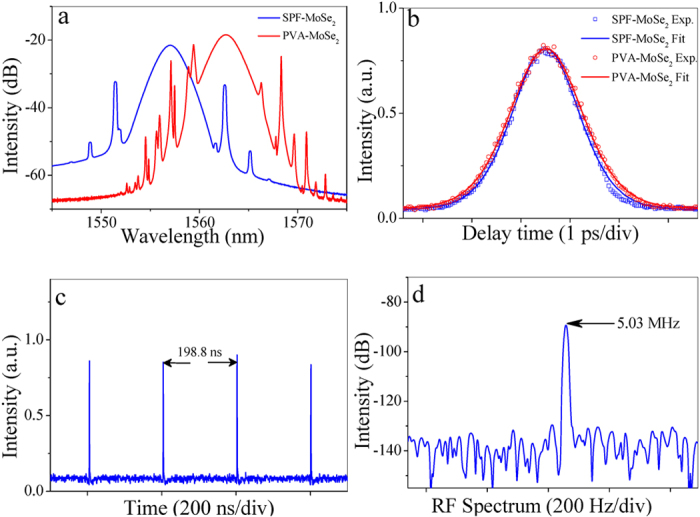
Output characteristics of MoSe_2_ mode-locked fiber laser. (**a**) Optical spectrum, (**b**) autocorrelation trace, (**c**) pulse train, and (**d**) RF spectrum.

By using SPF- and PVA-MoSe_2_ SAs, mode-locked operation can also be realized in the fiber laser, similar to that of WSe_2_ SA. The blue and red curves show the laser performances based on SPF- and PVA-MoSe_2_ SAs respectively, as demonstrated in Fig. 7. The spectral width, pulse duration, pulse interval, and fundamental repetition rate for SPF-MoSe_2_ mode-locked pulses are 2.3 nm, 1.09 ps, 198.8 ns, and 5.03 MHz, respectively. The corresponding time bandwidth product is calculated as 0.316, indicating that the output pulse is a chirp-free soliton. The central wavelength changes for different mode-locking operations, which is mainly attributed to the variation of pump power, insertion loss, and polarization state. Table 1 summarizes the SA parameters and the corresponding laser performance for a clear comparison.

E. M. Pessina *et al.* demonstrated that self-mode locking operation can be achieved due to multimode laser instability^49^. However, the instability-induced mode-locked fiber laser always operates at the fundamental repetition rate and cannot deliver stable picosecond pulses, which is different from our operations. For example, the MoSe_2_/WSe_2_ mode-locked fiber laser is capable of generating robust single/multiple pulses without average. Moreover, soliton mode locking operation could be also obtained in a fiber laser with length of 11 m using the proposed SAs. We have carried out comparative experiments to verify whether the mode locking operation is purely caused by WSe_2_/MoSe_2_ nanosheets. In the experiment, continuous wave is always observed in the fiber laser by using pure SPF (directly exposed on air), PVA film, or removing the WSe_2_/MoSe_2_ SAs, despite that the polarization controller and the pump power are tuned over a full range for hundreds of times. In contrast, mode-locking operation can be easily achieved by depositing the WSe_2_/MoSe_2_ nanosheets on SPF or inserting the PVA-WSe_2_/MoSe_2_ film into the laser cavity. The comparative results confirm again that the WSe_2_/MoSe_2_ SAs are responsible for the mode locking operation of the fiber laser.

## Discussion

We found that the dispersion setting and output ratio of fiber cavity strongly affect the laser operations. For example, mode-locking operation can be easily obtained at large anomalous dispersion regime with output ratio less than 10%. However, pulse operation is difficult to achieve at normal dispersion regime or fiber laser with output ratio larger than 20%. The results could be attributed to the small modulation of the WSe_2_/MoSe_2_ SAs. As the normal dispersion induced temporal broadening or output induced-perturbation cannot be compensated by the SA, mode-locking will not be established in the fiber laser. Experimental experiments show that MoSe_2_/WSe_2_ nanosheets exhibit saturable absorption at 1.55 μm (0.8 eV), although the photon energy is lower than the bandgap of the material. Several groups have proposed different theories to interpret the sub-bandgap saturable absorption, such as defect-induced bandgap decreasing^21^, coexistence of semiconducting and metallic states^22^, two-photon absorption saturation^24^, and edges state of the materials^50^. However, a unanimity interpretation of the governing mechanism has not been fully established. In the view of defect-state theory, the deviation from perfection in two-dimensional materials is inevitable and will renovate their electronic and optical properties^51^. By the introduction of sulfur defects in MoS_2_ or WS_2_, the bandgaps of these materials can be significantly reduced^21^,^25^,^52^,^53^, which may further contribute to the broadband saturable absorption. As these TMD materials possess similar lattice structures and photonic properties, we could deduce that sub-bandgap saturable absorption of WSe_2_/MoSe_2_ nanosheets may be similar with that of the MoS_2_ and WS_2_ reported previously.

## Methods

### Preparation of WSe_2_/MoSe_2_ nanosheets

**Figure 8:**
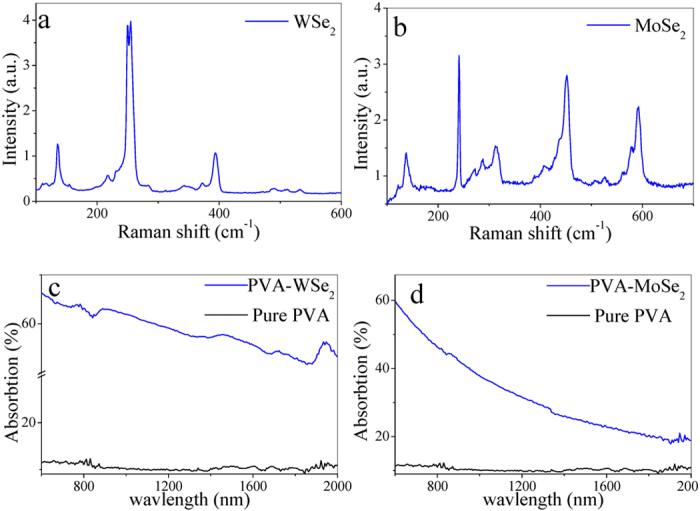
Raman spectra of (**a**) bulk WSe_2_ and (**b**) bulk MoSe_2_. (**c**) Linear absorptions of PVA-WSe_2_ film and (**d**) PVA-MoSe_2_ film in comparison with pure PVA-film.

Before the liquid exfoliation, we identify the WSe_2_/MoSe_2_ crystal by Raman spectroscopy using a 785 nm laser, as depicted in Fig. 8(a,b). The Raman spectra of bulk WSe_2_ and bulk MoSe_2_ agree well with the earlier findings^54^. The WSe_2_/MoSe_2_ nanosheets are prepared by a liquid exfoliation method that consists of three steps. First, a solvent is prepared by mixing 50 mg sodium deoxycholate bile salt surfactant with 5 mL deionized water. Indeed, the solvent must be elaborately chosen to match the surface energy of the layered materials. Then, a piece bulk WSe_2_/MoSe_2_ crystal (about 15 mg) is dropped into the prepared solvent, and the mixture is ultrasonic treated for 8 hours at power of 180 W. Note that impurities and defects are inevitable in the prepared sample, which may induce a small bandgap and thus a broadband optical response^21^. To remove the large aggregation, the dispersion is further centrifuged at 1000 rpm for 10 minutes and the 90% supernatant is collected for the next step of the experiment. The linear absorptions of PVA-WSe_2_ film, PVA-MoSe_2_ film, and pure PVA film are measured by a spectrometer (Hitachi UV4100). As shown in Fig. 8(c,d), the absorption coefficient decreases with the increase of wavelength from the visible to near-infrared band.

### Characterizing the nonlinear saturable absorption of WSe_2_/MoSe_2_ SA

**Figure 9:**
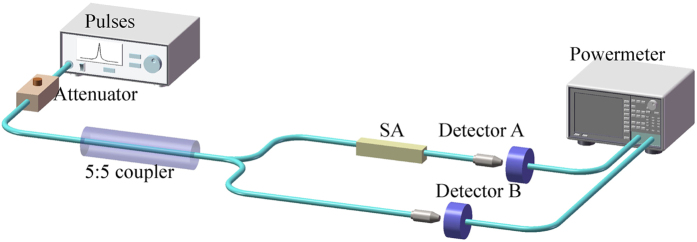
Schematic diagram of nonlinear optical characterization scheme.

The nonlinear saturable absorption property of SPF-WSe_2_ SA, PVA-WSe_2_ SA SPF-MoSe_2_ SA, and PVA-MoSe_2_ SA are investigated separately with a balanced twin-detector measurement scheme. The illumination pulse is generated from a 1.55 μm EDF laser with the pulse repetition rate of ~25 MHz and the duration of ~400 fs. As shown in Fig. 9, the illumination pulse is divided equally by a coupler and the test sample is inserted in one branch. By adjusting the intensity of illumination pulse, the transmission of the SA as a function of incident power is obtained by comparing the output powers of two branches.

## Additional Information

**How to cite this article**: Mao, D. *et al.* Erbium-doped fiber laser passively mode locked with few-layer WSe_2_/MoSe_2_ nanosheets. *Sci. Rep.* **6**, 23583; doi: 10.1038/srep23583 (2016).

## Figures and Tables

**Table 1:** Summarization of SA parameters and laser performance.

SA types	SA parameters		Nanosheets parameters		Mode-locked laser performance			
	Modulation depth (%)	Thickness (μm)	Size (μm)	Thickness (μm)	Central wavelength (nm)	Spectral Bandwidth (nm)	Pulse duration (ps)	Repetition Rate (MHz)
SPF-WSe_2_	0.3	//	0.4 ~ 1	~15	1556.7	2	1.31	5.31
PVA-WSe_2_	0.5	43.8			1557.6	2.1	1.25	5.31
SPF-MoSe_2_	1.4	//	0.4 ~ 1	~20	1557.1	2.3	1.09	5.03
PVA-MoSe_2_	0.4	24.9			1562.6	2.2	1.18	5.03
